# Nutrient Availability and Pathogen Clearance Impact Microbiome Composition in a Gnotobiotic Kimchi Model

**DOI:** 10.3390/foods14111948

**Published:** 2025-05-30

**Authors:** Devin H. Bemis, Carly E. Camphausen, Esther Liu, Joshua J. Dantus, Josue A. Navarro, Kieren Leif Dykstra, Leila A. Paltrowitz, Mariia Dzhelmach, Markus Joerg, Pamil Tamelessio, Peter Belenky

**Affiliations:** 1 Department of Molecular Microbiology and Immunology, Brown University, Providence, RI 02912, USA; devin_bemis@brown.edu; 2 Brown University, Providence, RI 02912, USA

**Keywords:** kimchi, *Escherichia coli*, *Bacillus cereus*, food safety, pathogen, microbial composition, lactic acid bacteria, fermentation

## Abstract

Kimchi is a fermented Korean food typically made with napa cabbage, garlic, radish, ginger, and chili pepper. It is becoming increasingly popular due to its flavor, high fiber content, and purported probiotic benefits. The microbial ecology of the fermentation community has been extensively studied, though what’s less understood is how its microbial community changes when nutrients or pathogens are introduced. To study this, we used gnotobiotic cabbage media inoculated with a kimchi starter culture as a model system. These inoculated samples were exposed to *E. coli* or *Bacillus cereus*, with or without added nutrients in the form of tryptic soy broth (TSB). We tracked pH, colony-forming units (CFUs), and community composition over time. We also used Oxford Nanopore sequencing to analyze the 16S rRNA gene (V4–V9), followed by use of the Emu algorithm for taxonomic assignments. As expected, LABs suppressed pathogens, but this effect was weaker early on in the nutrient-rich condition. Pathogen exposure changed the overall community—*Lactobacillus* species became more common, and *Leuconostoc mesenteroides* less so. Interestingly, adding nutrients alone caused similar microbial shifts to those seen with pathogen exposure. This could suggest that nutrient levels have a larger impact on the final microbiome structure than direct microbial competition. Together, these findings suggest that monitoring total microbial composition, and not just the presence of pathogens, may be important for ensuring kimchi fermentation reproducibility.

## 1. Introduction

The popularity of kimchi, a traditional Korean fermented cabbage dish, has grown in part due to increased interest in probiotics and gut health [1]. During kimchi preparation, cabbage is shredded, salted, and left to ferment for several days, primarily by LAB and yeast. This creates an acidic, selective microbial environment favorable to productive microbes, contributing to flavor and texture profiles [2,3,4]. Initial community composition depicts a highly diverse and dynamic microbial community, reflecting inoculation from microbes found in both the ingredients and the environment [3]. High salinity then selects for salt and aero-tolerant bacteria, which then consume available oxygen [5]. As the fermentation process transitions to anaerobic and acidic conditions, the microbial composition is also shaped through the production of antimicrobial peptides and other secondary metabolites [6]. The kimchi microbiome is ultimately dominated by LAB genera such as *Lactobacillus*, *Leuconostoc*, and *Weissella* [3,4]. Added sweeteners, such as fruits (pears, apples) and wheat or rice flour, increase free sugar content, further promoting LAB fermentation and refining microbial composition [7,8].

The fermentation process produces bioactive compounds (e.g., organic acids, bacteriocins) with antioxidant, anti-inflammatory, and microbiome-stabilizing properties [2,6,7,9,10]. In addition to health benefits, these metabolites create an environment resistant to foodborne pathogens such as *E. coli*, *B. cereus*, *Staphylococcus aureus*, and *Listeria monocytogenes* [9,11,12]. Despite this, *B. cereus* contamination has been documented in commercial kimchi, leading to food safety concerns and regulatory limits of 10,000 microbes/g set by the Korean Ministry of Food and Drug Safety (MFDS) [9]. Enterotoxigenic *E. coli* (ETEC) has similarly been detected in commercial kimchi, with examples of disease outbreaks tied to its consumption [13]. While fermentation is typically effective at eliminating *E. coli*, some studies report its survival in early fermentation stages. Recent research found that *E. coli* can be reduced within 1–2 days of kimchi fermentation [14], suggesting acidification and microbial competition may play a dominant role in suppression. However, this contrasts with earlier studies where *E. coli* O157:H7 persisted in acidic fermented foods for weeks [10,15]. These conflicting findings suggest that factors such as nutrient availability, fermentation temperature, and microbial composition may influence the speed and extent of *E. coli* clearance.

The presence of *E. coli* and *B. cereus* may affect kimchi’s microbial composition, pH, and metabolite production. Both *E. coli* and *B. cereus* prefer mesophilic, aerobic, neutral pH environments [16]. *B. cereus* also exhibits autolysis under high cell density, altered pH, and nutrient-limiting conditions [17]. The role of autolysis in pathogen reduction has not been fully explored, and it remains unclear whether nutrient availability influences the rate or extent of this process. As fermentation progresses, conditions shift outside the optimal growth range for these pathogens, typically leading to clearance.

Here, we investigate how kimchi’s antimicrobial properties affect the survival of pathogenic bacteria during fermentation. While the antimicrobial effects of bacteriocins (e.g., nisin) and LAB-driven acidification are known, the role of nutrient availability in pathogen inhibition remains unclear. Previous studies have also not probed the possibility of pathogen preference for nutrient rich environments, nor whether pathogen presence or nutrient supplementation impact kimchi’s final microbial composition. We sought to address these questions using a systematic gnotobiotic kimchi model allowing for controlled introduction of pathogens. Gnotobiotic Napa cabbage media [18] were inoculated with *E. coli* and *B. cereus*, alongside a kimchi starter culture, under varying nutrient conditions. Bacterial growth was assessed in three media types: pure cabbage media (CM), cabbage media supplemented with tryptic soy broth (TSB), and cabbage media buffered with Tris-HCl (THCL). TSB was selected not to mimic traditional kimchi ingredients, but rather to model a state of nutrient excess—providing a rich, undefined medium commonly used to study bacteria under high-nutrient conditions [19,20]. Colony-forming units (CFU) were measured via serial dilution plating at multiple time points to track *E. coli* and *B. cereus* survival. Additionally, microbial composition was analyzed using long-read 16S amplicon sequencing (V4–V9 regions) on an Oxford Nanopore Technologies MinION sequencer. Taxonomic abundance estimates were generated using the Emu algorithm [21] and the silva v138.1 SSU database. This approach allowed us to assess how the microbial composition was affected by pathogen interactions and nutrient supplementation with a more confident taxonomic classification. By integrating culture-based enumeration with high-resolution sequencing, this study provides new insights into how nutrient availability influences microbial competition, LAB dominance, and pathogen survival in the early stages of kimchi fermentation under laboratory conditions.

## 2. Materials and Methods

### 2.1. Media Preparation, Strains Used, and Culture Conditions

Cabbage media was prepared following the gnotobiotic cabbage media protocol as described previously [18]. Napa cabbage was blended with sterile water at a 2:1 water-to-cabbage ratio, then filtered through a standard coffee filter to remove large particulates. The resulting liquid was centrifuged to clarify the extract, and the supernatant was subsequently sterilized by passage through a 0.2 µm membrane filter. Sodium chloride was then added to achieve a final concentration of 2% (*w*/*v*) NaCl. To make the TSB and Tris-HCl media solutions, prepared cabbage media was supplemented with either tryptic soy broth (10X TSB) to make 100% TSB media or Tris-HCl (pH 7.5) to make a 10 mM final solution. The final media was re-sterilized using a 50 mL Steriflip^®^ (EMD Millipore Corporation, Billerica, MA, USA) filter unit.

*E. coli* strain MG1655 (ATCC 47076) was transformed with plasmid pUC19 to confer ampicillin resistance, cultured in Luria–Bertani broth (LB), mixed with 20% sterile glycerol and stored at −80 °C until use. *Bacillus cereus* (Frankland and Frankland; ATCC 11778) was cultured in TSB media and stored in the same fashion. The kimchi starter culture was prepared by straining the liquid portion of a semi-commercial kimchi fermentation (Soban, Providence, RI, USA) made with Napa cabbage and traditional ingredients, including garlic, ginger, scallions, chili powder, and fish sauce. The fermentation proceeded at room temperature until reaching a final pH of 3.6. After low-speed centrifugation, the liquid phase was filtered using an 8 µm filter to remove particulate matter. The filtered starter culture was mixed with 20% sterile glycerol and stored at −80 °C for experimental use.

### 2.2. Experimental Culture, CFU Quantification, and pH Measurement

Cabbage media (2% NaCl), with or without nutrient supplements, was inoculated with one of the following: (1) kimchi starter culture alone, (2) *E. coli* or *B. cereus* alone, or (3) a mixture of the kimchi starter with either pathogen. For the inoculations, 10 µL of starter or 100 µL of overnight bacterial culture was added to 10 mL of media. A total of 1.5 mL of each prepared mixture was dispensed into 24-well plates in triplicate. Plates were sealed with Breath–Rite film and incubated at 23 °C in the dark. Sample size was decided based on the innately low variability seen in the system during experimental development.

To sample microbial communities, the Breath–Rite film sealing each well was punctured with a sterile pipette tip. The culture was thoroughly mixed by pipetting, and 90 µL was withdrawn for CFU quantification. Serial dilutions (10^−1^ to 10^−^⁶) were performed in a 96-well plate using sterile saline as the diluent. From each dilution, 2 µL was plated in triplicate onto three selective media: LB agar with ampicillin (100 µg/mL) for *E. coli*, Mannitol Egg Yolk Polymyxin agar (MYP) for *B. cereus*, and MRS agar with vancomycin (10 µg/mL) for lactic acid bacteria (LAB). Droplets were applied in a non-overlapping grid and air-dried before incubating plates at 30 °C for 24 h.

pH was measured at each time point using a calibrated digital pH probe. The probe was submerged in the culture until readings stabilized. Between samples, the probe was cleaned with 10% bleach, rinsed with distilled water, and dried with lint-free tissue. CFU counts and pH values were recorded at 0, 3, 24, and 48 h of fermentation.

### 2.3. DNA Extraction, Amplification, Library Preparation, Sequencing

DNA was extracted from samples stored in DNA/RNA Shield using the ZymoBIOMICS Quick-DNA Fecal/Soil Microbe 96 Kit (Zymo D6011, Zymo Research Corporation, Irvine, CA, USA) following the manufacturer’s protocol. Total DNA was eluted into nuclease-free water. The 16S rRNA V4–V9 hypervariable regions were amplified from total DNA using the 515F forward primer from the Earth Microbiome Project30 (5′-GTGYCAGCMGCCGCGGTAA) paired with the U1492R universal reverse primer (5′-CGGCTACCTTGTTACGAC); using the 5X Phusion HF DNA Polymerase (Fisher Sci F530L, Fisher Scientific, Pittsburgh, PA, USA) in triplicate reactions. The amplification was performed on a Bio-Rad T100 Thermal Cycler under the following conditions: 98 °C for 3 min; followed by 35 cycles of 98 °C for 45 s, 50 °C for 60 s, and 72 °C for 90 s, and a final extension at 72 °C for 10 min. Triplicates were reconsolidated into single tubes before the library preparation steps. Amplicons were barcoded using the Native Barcoding 24 v14 kit (ONT SQK-NBD114-96, Oxford Nanopore Technologies, Oxford, UK) and sequenced on the Oxford Nanopore Technologies MinION MK1B (ONT MIN-101B) following the manufacturer’s protocol.

### 2.4. Computational Analysis

Sequencing reads were basecalled, demultiplexed, and adaptors were trimmed, all using Oxford Nanopore Technologies’ Guppy Software (version 6.5.7). Taxonomic abundance estimates were generated using the Emu algorithm [21] (version 3.4.5) and the SILVA SSU database (version 138.1). Rarefaction of counts was not performed, due to recommendations against this practice [22]. Data were formatted using the R (version 4.3.2) packages tidyverse (version 2.0.0) and phyloseq (version 1.24.2) to make relative abundance barplots. Relative abundance for each taxon was averaged across replicates (*n* = 3). Differentially abundant taxa were identified using DESeq2 [23] (version 1.40.2; significance threshold of adj-*p* < 0.05) and visualized using ggplot2 (version 3.5.2). Adjusted *p*-values were computed using the Wald test with Benjamini–Hochberg correction. Volcano-plot labels were modified with ggrepel (version 0.9.6). CFU and pH plots were made with GraphPad Prism (version 10.3.1). The Principal Coordinate Analysis plot was made using the Bray–Curtis dissimilarity metric.

## 3. Results

### 3.1. LAB-Mediated Inhibition of Pathogens Depends on pH and Nutrient Availability

To assess how pathogenic bacteria respond to the presence of a LAB starter culture—and how their presence, in turn, alters the microbial community structure—we conducted a series of fermentation experiments under different nutrient and pH conditions. Specifically, we aimed to investigate how nutrient availability and pH buffering affect the growth dynamics of LAB and the survival of foodborne pathogens *E. coli* and *B. cereus*. To do this, we modified cabbage fermentation media [18] across three conditions: a control cabbage media containing 2% NaCl, a cabbage media containing 1X TSB, and a cabbage media buffered with 10 mM Tris-HCl (pH 7.5). Changes in pH and CFU were measured over a 48 h time frame to evaluate microbial interactions and competitive dynamics.

**Figure 1:**
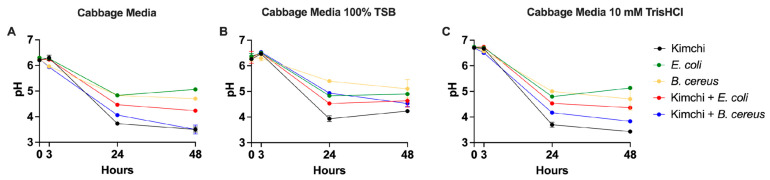
(**A**) pH levels for the pure cabbage media (CM) at time points 0, 3, 24, and 48 h post-inoculation with kimchi starter; (**B**) pH levels for the cabbage media supplemented with tryptic soy broth (TSB) at time points 0, 3, 24, and 48 h post-inoculation with kimchi starter; (**C**) pH levels for the cabbage media buffered with Tris-HCl (THCL) at time points 0, 3, 24, and 48 h post-inoculation with kimchi starter. Error bars represent standard deviation (*n* = 3).

Across all conditions, we noted that the pH dropped over the 48 h incubation period; however, the rate of change and terminal pH were impacted by the microbial and media composition (Figure 1A–C). The starting pH was the same for the control and the TSB condition (6.2–6.3), whereas the buffered media had an elevated initial pH (6.7), as expected. At three hours, none of the conditions led to a discernible change from the initial pH. At 24 h, the pH of all the communities dropped (3.6–5.4), with the most dramatic decreases occurring in the kimchi starter culture (LAB-dominated) conditions (3.6–4.0). The addition of either one of the two pathogens to the kimchi in all media conditions led to a higher pH (4.0–5.0), and the pathogens incubated without the kimchi starter had the highest 24 h pH in each group (4.8–5.4) (Figure 1A–C). Interestingly, the TSB media had a higher 24 h and 48 h pH than the buffered or the CM condition (3.8–5.4; 3.4–5.2; 3.3–5.1, respectively). This indicates that the higher-nutrient-content media inhibited acidification.

The pathogen-challenged kimchi conditions exhibited pH dynamics comparable to their unchallenged counterparts across both CM- and TSB-supplemented media. However, by 48 h, the differences between treatment groups in the TSB condition (total range of 4.2–5.4) were less pronounced than in CM (total range of 3.3–5.1), with all samples converging to similar pH levels. This convergence suggests that high nutrient availability in TSB may dampen the acidifying capacity of LAB, possibly by favoring broader microbial growth that competes for resources or interferes with lactic acid production. In the THCL media, pH trends mirrored those of the other conditions, though initial pH values were consistently higher due to buffering. By 48 h, however, buffering effects diminished, and pH values aligned closely with those of unbuffered CM, indicating the limited long-term impact of pH stabilization on fermentation dynamics.

**Figure 2:**
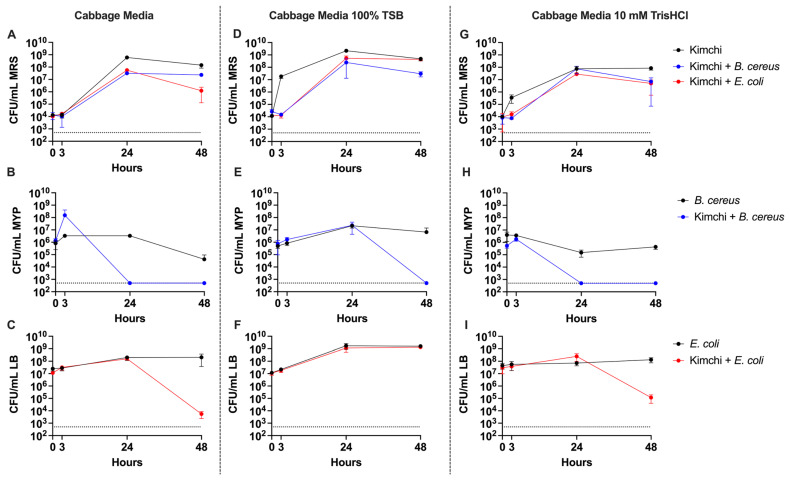
Quantification of lactic acid bacteria CFU/mL on MRS agar, *B. cereus* CFU/mL on MYP, and *E. coli* CFU/mL on LB agar, from time points 0, 3, 24, and 48 h post-inoculation in (**A**–**C**) CM, (**D**–**F**) TSB-supplemented CM, and (**G**–**I**) CM buffered with 10 mM Tris-HCl. Error bars represent standard deviation (*n* = 3) and the dotted-line represents the limit of detection of 500 CFU/mL.

We also quantified the total LAB CFU by plating on De Man–Rogosa–Sharpe (MRS) agar supplemented with Vancomycin, *E. coli* CFU by plating on LB supplemented with Ampicillin (AMP), and *B. cereus* CFU by plating on Mannitol Egg Yolk Polymyxin (MYP) agar. Across all media conditions, the CFU of LAB in unchallenged samples peaked at 24 h, after which they plateaued or decreased by 48 h, indicating that the initial 24 h was sufficient to establish LAB dominance (Figure 2A–C). Notably, in the TSB-supplemented condition, LAB growth appeared to accelerate earlier, with a substantial increase in CFU observed between the 0 h and 3 h time points, suggesting that nutrient enrichment may initially promote faster LAB proliferation. However, in each condition challenged with either *E. coli* or *B. cereus*, the maximum LAB CFU were mostly reduced. This reduction indicates that competitive pressure from pathogen co-inoculation may limit both the rate of LAB expansion and the total population size achieved during fermentation.

To evaluate whether LAB could suppress high pathogen loads, we challenged the fermentation with *B. cereus* and *E. coli* at levels two orders of magnitude higher than the LAB inoculum. *B. cereus* was particularly sensitive to LAB-mediated inhibition, dropping below the limit of detection by 24 h in both CM and THCL media (Figure 2E). Interestingly, in TSB media, *B. cereus* persisted through the 24 h mark, suggesting that additional nutrients conferred protection—potentially by preventing autolysis or buffering against acidification. Despite this transient protection, *B. cereus* levels still fell below detection by 48 h.

*E. coli* showed a similar trend of LAB-dependent inhibition but with delayed clearance. Significant reductions in CFU were not observed until between 24 and 48 h in both cabbage and buffered media. In the TSB condition, however, *E. coli* counts remained relatively stable, indicating that inhibition was attenuated, likely due to the elevated pH rather than hypothetical protection from autolysis, which is less relevant to *E. coli*. These results suggest that while unenhanced cabbage media supports strong LAB-mediated inhibition of pathogens, nutrient-rich fermentations, such as those supplemented with fruit or rice [7], may reduce this protective effect.

### 3.2. Distinct Fermentation Microbiomes Shaped by Media Composition and Pathogen Challenge

To identify the bacteria present at both the genus and species level in each of the three experimental media (CM, TSB, THCL), as well as in the kimchi starter culture (Soban), we used Oxford Nanopore sequencing (V4–V9 16S rRNA) followed by Emu taxonomic classification. Our first assessment was how well the artificial culture conditions replicated the starter culture after 48 h (Figure S1A,B). None of the experimental media accurately replicated the Soban traditional kimchi microbiome. While TSB exhibited the closest resemblance at the genus level, with *Lactobacillus* and *Leuconostoc* as dominant genera (Figure S1B), species-level analysis revealed significant discrepancies. Specifically, *L. mesenteroides* dominated CM and THCL despite its low abundance in the Soban culture (Figure S1A). TSB also showed dominance by *L. mesenteroides* but contained notable levels of *Lactobacillus curvatus* and *Lactobacillus sakei*, species prevalent in the starter. However, key starter species, including *Leuconostoc gelidum*, *Leuconostoc miyukkimchii*, and *Leuconostoc carnosum*, were absent from all experimental media. This initial analysis also revealed two limitations of the Nanopore sequencing pipeline: difficulty resolving closely related species at the species level and barcode leakage between samples [24], evidenced by the detection of low levels of pathogens in non-challenged controls.

**Figure 3:**
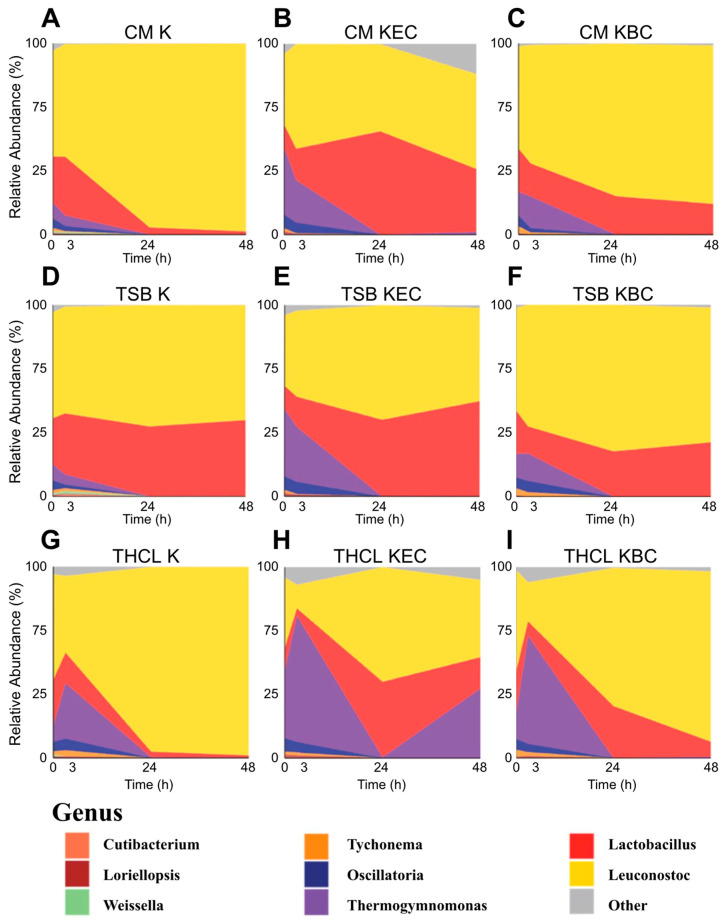
Relative abundances of top 8 bacterial genera from each media treatment group (**A**–**C**) CM, (**D**–**F**) TSB, (**G**–**I**) THCL, averaged from 3 replicates at time points 0, 3, 24, and 48 h. All other bacterial genera are shown in gray. Genera with pathogenic species are excluded.

In the pathogen-challenged conditions (Figure S2), *Escherichia* and *Bacillus* genera remained abundant throughout the time course, despite CFU data (Figure 2) indicating their elimination by 48 h. This discrepancy likely reflects the persistence of DNA from non-viable cells—a known limitation of amplicon sequencing, which can detect dead bacteria and thus overestimate their abundance. In Figure 3 and all subsequent analyses, we addressed this by computationally removing *Escherichia* and *Bacillus* sequences, allowing clearer interpretation of LAB community dynamics. Additionally, the low-level presence of pathogens in uninoculated controls suggests minor barcode leakage—a known limitation of Nanopore sequencing [24]—likely amplified in low-biomass samples such as T0 starter cultures.

We found that across all treatments, *Leuconostoc* dominated by 48 h, and the increases in relative abundance of *Leuconostoc* and *Lactobacillus* appeared to correlate with a decline in *Thermogymnomonas*. *Thermogymnomonas* dropped to near-zero relative abundance by 24 h in all treatments except THCL challenged by *E. coli* (Figure 3H). CM pathogen-challenged communities showed an increased relative abundance of *Lactobacillus* compared to the unchallenged condition. TSB cultures followed a similar pattern while potentially supporting greater retention of *Lactobacillus* by 48 h than CM or THCL, likely due to the broader availability of nutrients. THCL conditions exhibited a distinct *Thermogymnomonas* response compared to the other two media formulations: both challenged and unchallenged samples showed a pronounced spike at 3 h, suggesting that buffering may have influenced early-stage bacterial competition, an effect that disappeared by 24 h. While THCL and THCL + *B. cereus* (Figure 3G,I, respectively) were dominated by *Leuconostoc* at 48 h, the THCL + *E. coli* condition had a second spike of *Thermogymnomonas*, reducing *Leuconostoc* to less than half the total abundance. Interestingly, all media conditions challenged by *E. coli* (Panels B, E, H) showed comparable final *Leuconostoc* levels relative to other dominant genera, including *Lactobacillus*, *Thermogymnomonas*, and others. These results suggest that TSB may help preserve key community members like *Lactobacillus*, while pH buffering alters the community’s early response to pathogen challenge in a medium-specific manner.

**Figure 4:**
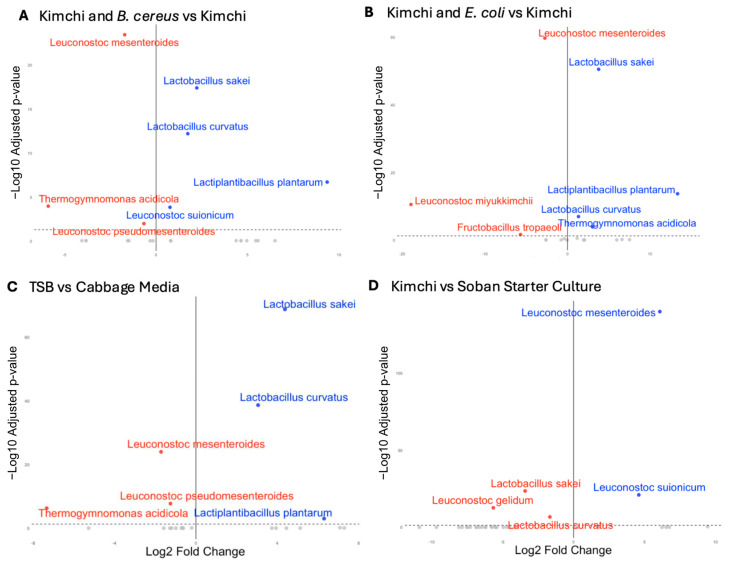
(**A**,**B**) Differential abundance of bacterial species in KBC and KE inoculated samples compared to unchallenged kimchi samples (*n* = 3). Abundance differences shown on a Log2 fold change scale. Significance of change between conditions determined by DESeq2 calculated adjusted *p*-values (threshold adj-*p* < 0.05). (**C**) Differential abundance of bacterial species in TSB media samples compared to pure cabbage media samples. (**D**) Differential abundance of bacterial species in inoculated samples compared to starter culture composition. The dotted-line represent the significance threshold (adj-*p* < 0.05). Species names in blue are found in greater relative abundance, and red less.

To determine the statistical relevance of the taxonomic observations described above, we used DESeq2 [23] to identify significant differences in microbial compositions at the 48 h time point. First, we analyzed the impact of challenging kimchi starters with *B. cereus* across all three medium types (Figure 4A). We identified that *Lactobacillus plantarum*’s relative abundance was 654-fold higher in the challenged samples (Figure S3). *L. sakei* and *L. curvatus* also had greater relative abundance, with 4.68 and 3.33-fold increases, respectively. On the other hand, *L. mesenteroides* had a marked decrease in relative abundance, with 3.28 less relative abundance in the *B. cereus* treated samples than in kimchi starter culture alone. This shift may reflect the influence of *B. cereus*–produced antimicrobial compounds, which could differentially affect community members—potentially limiting *Leuconostoc* while enabling more resilient *Lactobacillus* species to expand [25,26].

Next, we examined the effect of an *E. coli* challenge across media conditions. We identified taxonomically similar but more drastic differences in relative species abundance compared to challenging kimchi starters with *B. cereus*. In the *E. coli* challenged samples, *L. sakei* had a relative abundance 13.6-fold higher and *L. plantarum* 10,300-fold higher than that of the unchallenged kimchi samples, while *L. mesenteroides* was 6.67 times less as relatively abundant when compared to kimchi controls. This shows a similar pattern to Figure 4A, where a pathogen challenge may lead to an advantage for other LABs over *Leuconostoc.*

TSB-supplemented media conditions, as compared to CM, led to similar taxonomic abundance patterns as challenging the starter with pathogenic bacteria (Figure 4A). Compared to control cabbage media, samples inoculated in TSB media had a 20.7-fold increase in relative abundance of *L. sakei*, 8.3-fold greater *L. curvatus*, and 78.7-fold greater *L. plantarum*, while *L. mesenteroides* had a 3.3-times lower relative abundance in TSB samples (Figure S5). The buffered media led to no significant species-level differences in bacterial composition compared to CM, likely due to the ineffectiveness of the buffer by the 48 h time point.

Finally, we compared the 48 h CM samples without the pathogen challenge to the starter culture (Figure 4D and Figure S3). Compared to the starter culture, 48 h samples had a 67.5-fold greater relative abundance of *L. mesenteroides*, 10.6-times lower relative abundance of *L. sakei*, and 3.3-times lower relative abundance of *L. curvatus*. This link suggests a greater compositional similarity between starter culture and pathogen-unchallenged samples, with unchallenged samples appearing closer to 2/3 starter culture samples in a Principal Coordinate Analysis plot based on Bray–Curtis dissimilarity (Figure S6).

## 4. Discussion

This study profiled how nutrient availability and microbial competition shape the survival of foodborne pathogens during the early stages of kimchi fermentation. LAB effectively suppressed *E. coli* and *B. cereus* in low-nutrient and acidic environments. However, nutrient-rich media delayed clearance and shifted LAB dynamics, highlighting that fermentation conditions influence microbial composition and pathogen inhibition.

The ability of LAB to clear pathogens varied noticeably with media composition: plain cabbage media allowed for rapid acidification and effective inhibition, while pH-buffered conditions briefly delayed this effect without changing the final outcome. In contrast, nutrient-rich conditions (TSB) noticeably slowed LAB dominance and prolonged pathogen survival. This slower clearance seen in TSB media may be due to its high nutrient content, which supports the growth of both LAB and pathogens, reducing the competitive edge typically held by LAB in more limited environments. Also, in nutrient-rich conditions, we hypothesize that *B. cereus* may avoid autolysis due to ample resources, while *E. coli* remains viable for longer because acidification is delayed. In the context of *E. coli*, the acidification of the media is likely not the only cause of CFU reduction, since recent research shows that *E. coli* is tolerant of acidic conditions for much longer than 48 h [10]. Although TSB accelerated early LAB growth, this may have promoted broader microbial competition, interfering with LAB’s acidifying dominance. Despite these differences in clearance rates, the final microbial composition still resembled typical kimchi communities. This suggested that fermentation can proceed robustly even when challenged—though nutrient content may affect food safety dynamics in the early stages.

We also evaluated whether the gnotobiotic cabbage media [18] supported the full microbial diversity of the kimchi starter culture. At 48 h, the unchallenged cabbage media did not fully recapitulate the starter’s bacterial taxonomy and was dominated by *L. mesenteroides* to the near exclusion of other species—underscoring the simplification inherent to our model. This limited diversity may be explained by oxygen availability, nutrient content, incubation time, or a combination of these factors. Several species present in the starter but absent from our experimental media—*Leuconostoc gelidum*, *Leuconostoc miyukkimchii*, *Leuconostoc kimchii*, and *Leuconostoc carnosum*—are facultative anaerobes that may be more competitive under low-oxygen conditions [27,28,29]. It is also possible that the aerobic conditions in 24-well plates did not fully replicate the anaerobic environment of late-stage kimchi fermentation. We did, however, observe an expansion of the less aerotolerant *L. mesenteroides*, suggesting that oxygen levels decreased over time. Follow up experiments could investigate the use of deeper culture wells, or even anaerobic incubation. Interestingly, the addition of TSB modestly improved microbial diversity, bringing the community structure closer to that of the starter culture. This suggests that nutrient limitation in plain cabbage media may restrict the growth of certain species—particularly relevant since traditional kimchi recipes often include sugar and amino acid-rich supplements like pear and fish sauce. In fact, the final microbial profile in the cabbage-only media more closely resembled that of sauerkraut [30], a fermentation that solely relies on cabbage-derived nutrients. Finally, our incubations lasted only 48 h, and while it is possible that longer fermentation could have led to further shifts in microbial composition, the early peak in LAB populations by 24 h (Figure 2A–C) suggests that the community had already reached a relatively stable state by that point. Subsequent work could involve sampling from later timepoints to capture final stages in the sere.

In addition to nutrient availability, the presence of pathogens also influenced community composition. Both *E. coli* and *B. cereus* led to substantial increases in the relative abundance of *Lactobacillus* species, particularly *L. plantarum* (10,300- and 654-fold, respectively) and *L. sakei* (13.6- and 4.68-fold). This suggests that these pathogens may alter environmental conditions, such as nutrient availability or competitive interactions that selectively favor the growth of certain *Lactobacillus* taxa. These species are known for their acid tolerance and robust responses to stress, which may explain their competitive advantage in pathogen-altered environments [31]. The ability of certain LAB genera to diversify their metabolic repertoire may confer a competitive advantage under challenging or nutrient-poor conditions [32,33].

Interestingly, *L. mesenteroides*, which typically dominates the early stages of kimchi fermentation [3], was consistently reduced across all pathogen treatments. This decline across the board suggests that *Leuconostoc* may be more sensitive to the environmental changes induced by pathogen presence, possibly due to lower tolerance to antimicrobial peptides, competition for nutrients or low pH. This aligns with other studies which have found that *L. mesenteroides*’ poorer pH tolerance results in growth stalling around a pH of 5.4 to 5.7, whereas that of *L. plantarum* stalls around 4.6 to 4.8, explaining the differential growth rates [34].

Furthermore, the similarity between the microbial shifts observed in pathogen-inoculated samples (increased relative abundance of *L. sakei*, *L. curvatus*, *L. plantarum*, and reduction in *L. mesenteroides*) and those in the TSB-treated control also supports the idea that changes in available nutrients, rather than direct antagonism, may drive community restructuring. Similarly, in these TSB-supplemented cultures, *B. cereus* and *E. coli* survival was prolonged, further pointing to the large impact of nutritional availability on pathogen survival.

Our findings provide insights into microbial dynamics in kimchi fermentation, but several methodological limitations must be considered in the interpretation of results. This study had a relatively low sample size, which can limit statistical power in microbial community studies. However, we observed minimal variability in pH, CFU counts, and overall microbial composition across replicates, suggesting that this limitation likely did not impact the reliability of our findings. Similarly, only one microbial starter culture was used for consistency; a different initial population of LAB could impact factors, such as pathogen inhibition, and nutrient utilization. Future studies might use several starter cultures of distinct origin in parallel, as well as a mixed starter. Broader sampling might help findings be more widely applicable. Additionally, we observed minor barcode leakage between samples—a known limitation of Oxford Nanopore sequencing [24]—which may have contributed to the low-level detection of pathogens in uninoculated controls. Our use of long-read 16S rRNA sequencing allowed for improved taxonomic resolution, and we were able to confidently assign many community members to the species level—an advantage over traditional short-read approaches. Though even with long-read data, closely related bacterial species remained difficult to distinguish due to their high sequence similarity. For instance, *Bacillus cereus*, *Bacillus anthracis*, and *Bacillus thuringiensis* share 96.5% amino acid identity and are considered genetically indistinguishable [35]. Likewise, *E. coli* displays 99.9% and 99.8% 16S sequence similarity to *Shigella sonnei* and *Shigella flexneri*, respectively, and 99.6% similarity to *Salmonella enterica* serovar Enteritidis ES22 [36]. These taxonomic ambiguities limited our ability to resolve certain groups at the species level, requiring genus-level classification in some cases. In our results, we saw evidence of DNA from non-viable cells in our sequencing data which we corrected for based on CFU data, however no culture-independent viability assays were used to validate these findings such as performing qPCR or live/dead staining of cells. Findings were also limited to compositional changes between conditions, without any insight into functional properties and differences leading to these changes. To validate purported mechanisms of pathogen inhibition, continued experiments will look to use metabolomic analysis to link compositional changes to functional alterations.

In sum, we found that *B. cereus* and *E. coli* are largely inhibited by day two of kimchi fermentation, yet their transient presence has a measurable impact on the structure of the microbial community—promoting the growth of acid-tolerant Lactobacillus species while suppressing key fermentative microbes like *L. mesenteroides*. These findings reinforce why fermented vegetables like kimchi have a strong inherent resistance to foodborne pathogens: rapid acidification and competitive microbial ecosystems act as robust barriers to long-term pathogen survival. Our results also suggest that even short-lived pathogen presence can reshape the fermentation trajectory—without necessarily leaving pathogens detectable in the final product. This has important implications for how we assess and monitor food quality; measuring pathogen load alone may not be sufficient. Microbial composition itself can serve as a sensitive indicator of prior contamination or suboptimal fermentation conditions, and may be critical for maintaining product quality.

## Data Availability

The data presented in this study are openly available in the NCBI’s Sequence Read Archive (SRA) under BioProject Accession: PRJNA1256889; ID: 1256889.

## Supplementary Materials

The following supporting information can be downloaded at: https://www.mdpi.com/article/10.3390/foods14111948/s1, Figure S1: Stacked Composition Barplots from 48-h Timepoint Samples; Figure S2: Compositional Area Plots at Genus-Level Across Timepoints; Figure S3: Boxplots of Differential Relative Abundance at Species-Level Between Inocula; Figure S4: Boxplots of Differential Relative Abundance Species-Level from Starter; Figure S5: Boxplots of Differential Relative Abundance Species Between Media; Figure S6: Bray-Curtis PCoA Graph from 48-h Timepoint Samples.
