# Supplementary material for: Nutrient Availability and Pathogen Clearance Impact Microbiome Composition in a Gnotobiotic Kimchi Model
Source: Foods. 2025 May 30;14(11):1948. doi: 10.3390/foods14111948 (PMC12155097; doi:10.3390/foods14111948)
Supplement: Supplementary file 1 [file foods-14-01948-s001.zip › foods-3631314-supplementary.pdf]

Supplementary Figures: Nutrient Availability and Pathogen Clearance Impact Microbiome Composition in a Gnotobiotic Kimchi Model

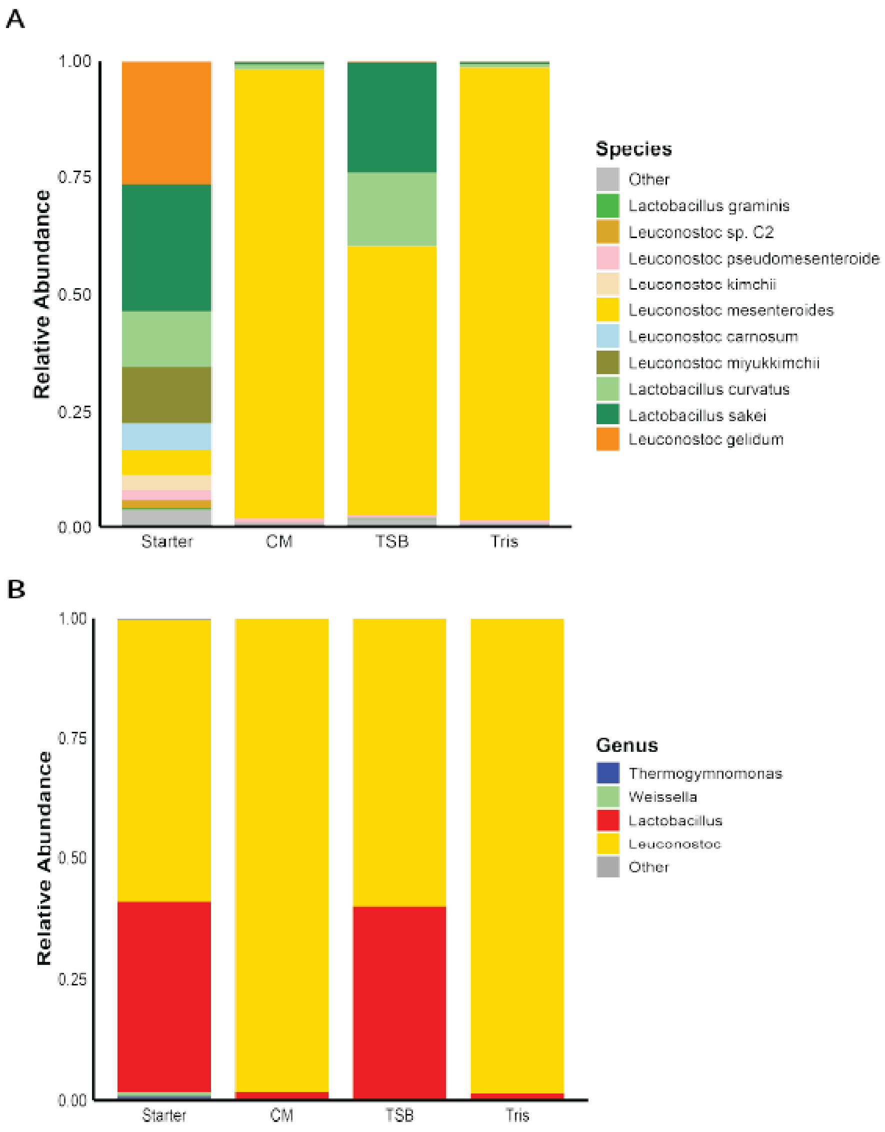

Supplementary Figure S1. Microbial (**A**) species and (**B**) genera from each media treatment group (CM, TSB, THCL) 48 hours post-inoculation (n = 3), alongside the un-incubated starter culture (n = 3).

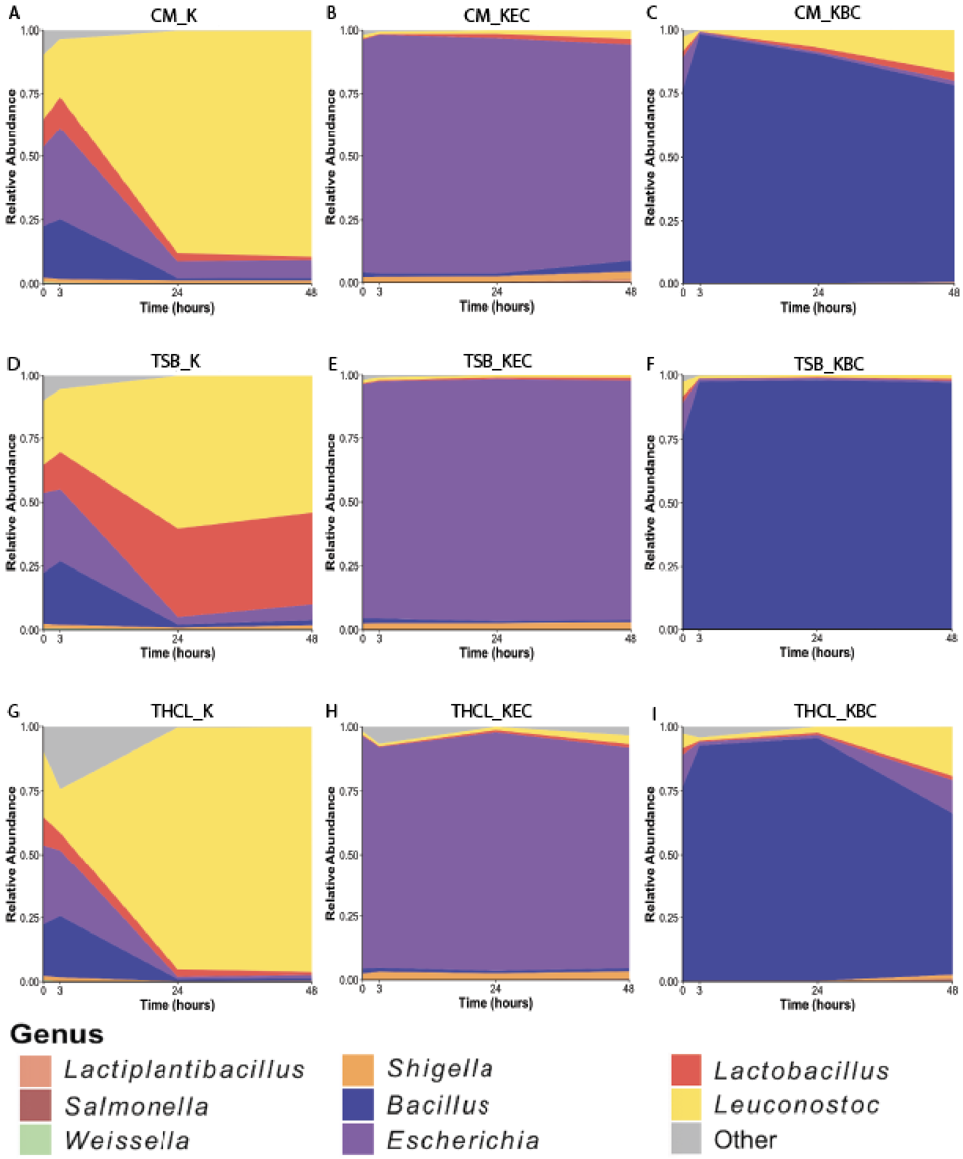

Supplementary Figure S2. Area plots displaying the relative abundance of the 8 most prevalent bacterial genera identified in the Cabbage Media (**A-C**), Cabbage Media 100% TSB (**D-F**), and Cabbage Media Tris-HCL (**G-I**) samples at time points 0h, 3h, 24h, and 48h. Unchallenged kimchi starter (K) and pathogen-challenged kimchi starter (KEC, KBC) are included. (n = 3).

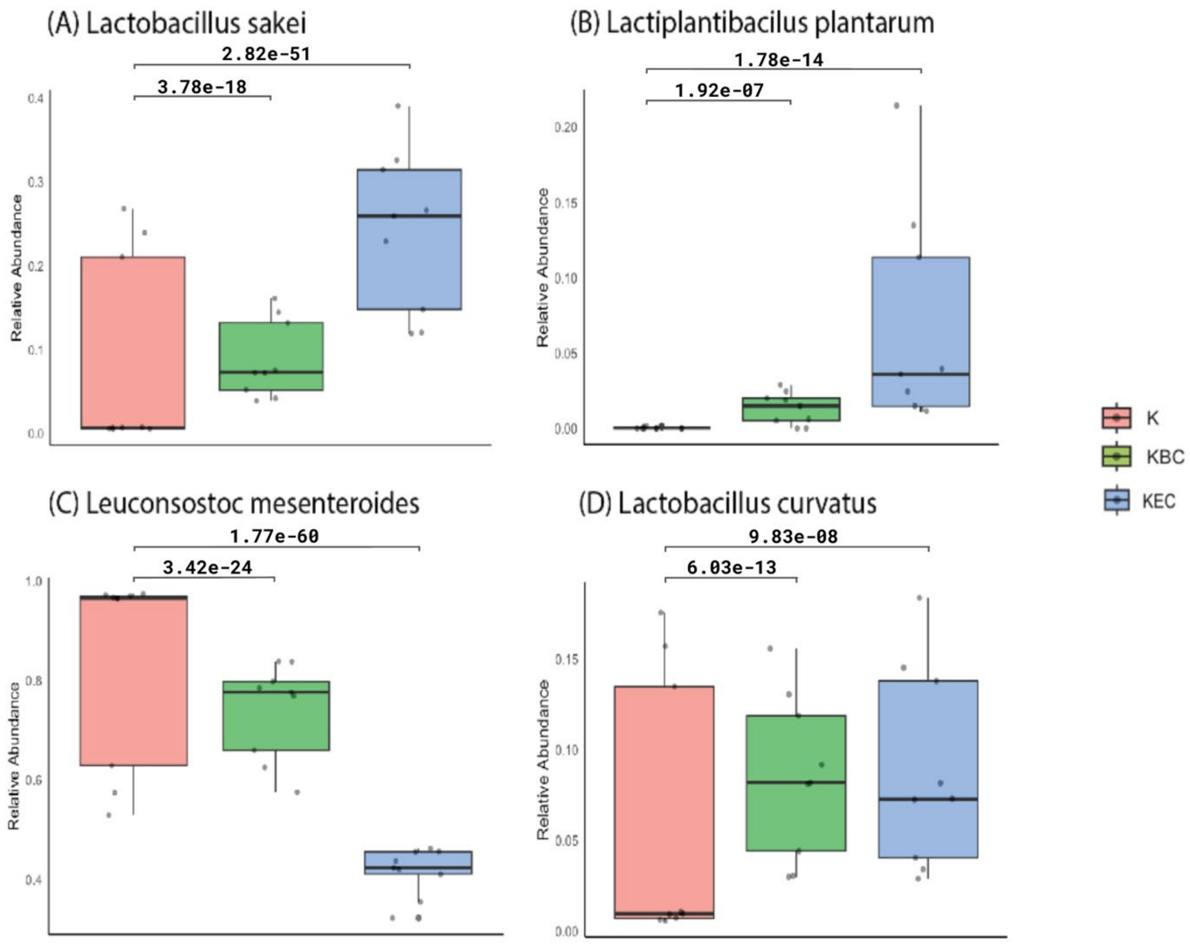

Supplementary Figure S3. Relative abundance of (**A**) *L. sakei*, (**B**) *L. plantarum*, (**C**) *L. mesenteroides*, and (**D**) *L. curvatus* across different inoculum types: Kimchi (K), Kimchi with *B. cereus* (KBC), and Kimchi with *E. coli* (KEC) across all media conditions at 48 hrs ($n = 9$). Boxes represent the interquartile range (IQR) with a line at the median, and whiskers extending to the minimum and maximum values. Adjusted p-values were computed using the Wald test with Benjamini-Hochberg correction.

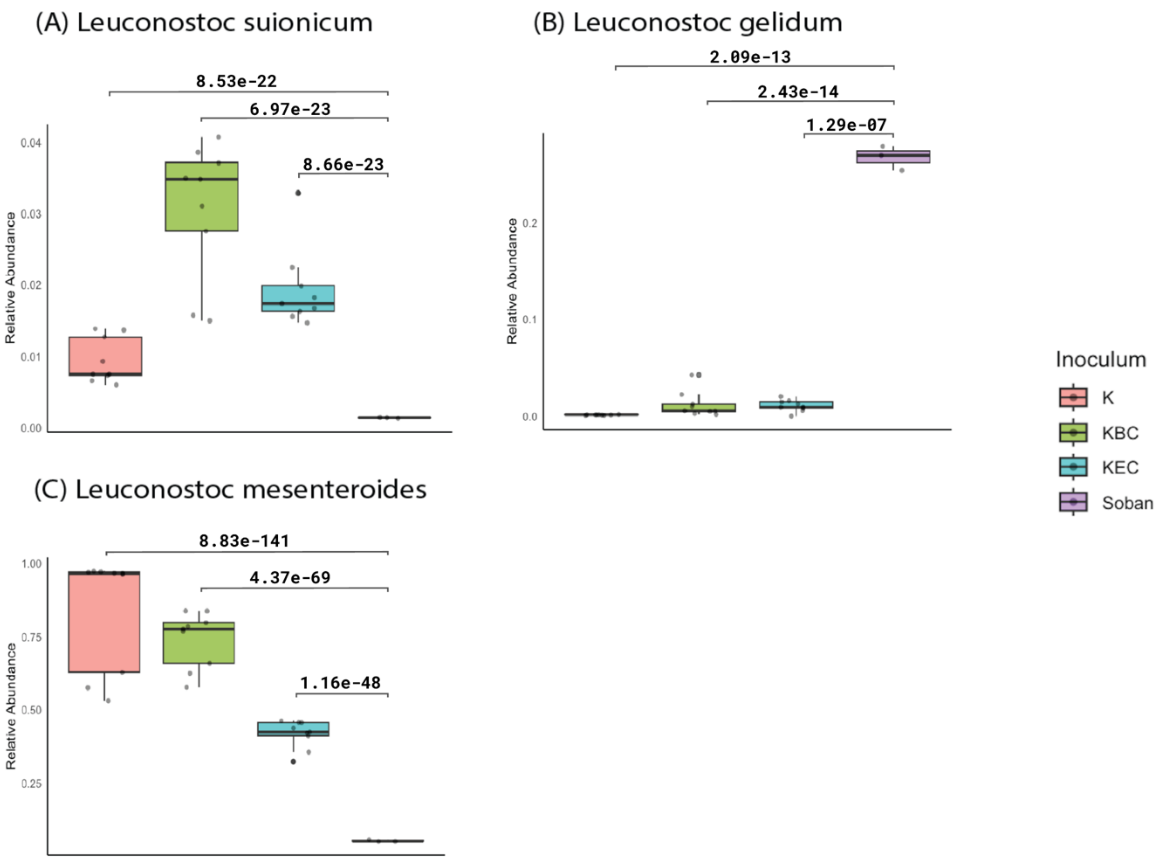

Supplementary Figure S4. Relative abundance of (**A**) *L. suionicum*, (**B**) *L. gelidum*, and (**C**) *L. mesenteroides* across three inocula at the 48-hour timepoint (n = 9) compared to relative abundance in the kimchi starter culture (Soban; *n* = 3). Boxes represent the interquartile range (IQR) with a line at the median, and whiskers extending to the minimum and maximum values. Adjusted p-values were computed using the Wald test with Benjamini-Hochberg correction.

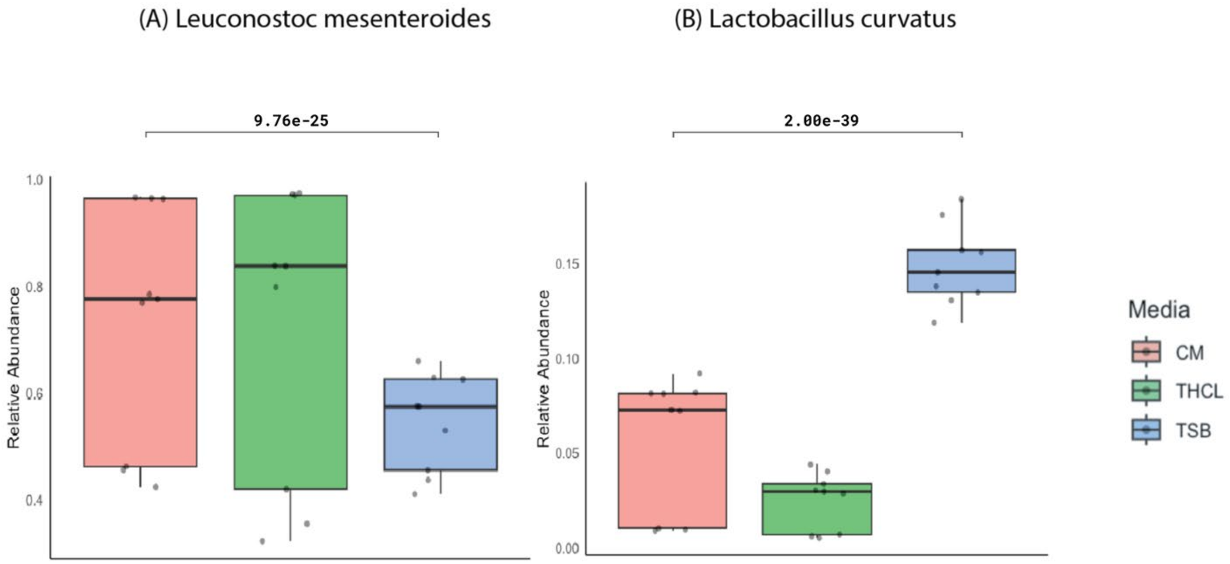

Supplementary Figure S5. Relative abundance of (**A**) *L. mesenteroides* and (**B**) *L. curvatus* across three media types (CM, THCL, TSB) at the 48-hour timepoint (*n* = 9). Boxes represent the interquartile range (IQR) with a line at the median, and whiskers extending to the minimum and maximum values. Adjusted p-values were computed using the Wald test with Benjamini-Hochberg correction.

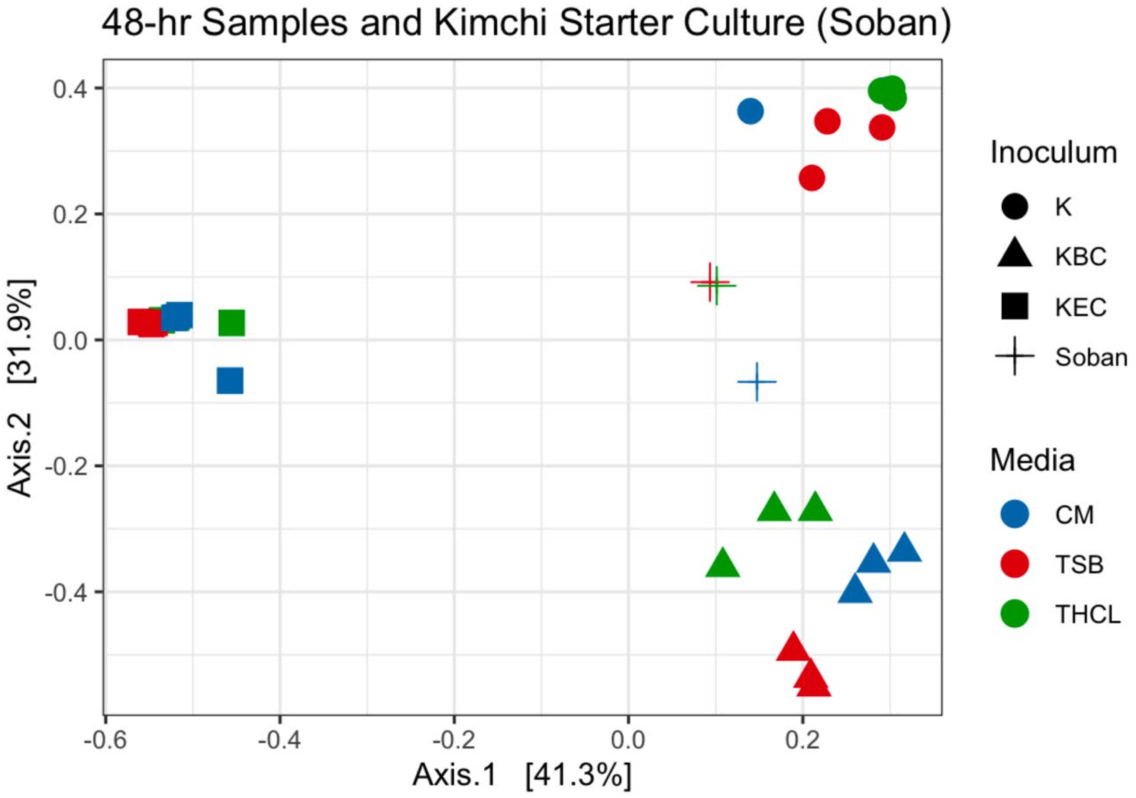

Supplementary Figure S6. Principal Coordinate Analysis (PCoA) graph based on Bray-Curtis dissimilarity, showing 48-hr timepoints from each condition and the starter culture (Soban).
